# The Inhibitory Effect of 3**β**-Hydroxy-12-oleanen-27-oic Acid on Growth and Motility of Human Hepatoma HepG2 Cells through JNK and Akt Signaling Pathway

**DOI:** 10.1155/2013/685159

**Published:** 2013-11-27

**Authors:** Juanjuan Wang, Xiangfeng Chen, Zhihua Zhou, Jinhui Li, Hongxiang Sun

**Affiliations:** ^1^Key Laboratory of Animal Virology of Ministry of Agriculture, College of Animal Sciences, Zhejiang University, Hangzhou 310058, China; ^2^Analysis Center of Agrobiology and Environmental Sciences, Zhejiang University, Hangzhou 310058, China

## Abstract

3**β**-Hydroxy-12-oleanen-27-oic acid (ATA) was a main antitumor active triterpene from the rhizomes of *Astilbe chinensis*. In this study, we investigated its effects on growth, apoptosis, cell cycle, motility/invasion, and metatasis in human hepatoma HepG2 cells *in vitro* and antimetastasis of B16-F10 melanoma in mice *in vivo*, as well as its molecular mechanisms of action using a high-throughput Cancer Pathway Finder PCR Array. ATA could not only induce tumor cells into apoptosis through the activation of both extrinsic and intrinsic pathways, arrest HepG2 cells in G_2_/M phase, but also suppress the invasion and metastasis abilities of HepG2 cells and the lung metastasis of B16-F10 melanoma in mice. PCR array assay revealed that ATA upregulated 9 genes including CDKN1A, MDM2, CFLAR (CASPER), TNFRSF10B (DR5), c-Jun, IL-8, THBS1, SERPINB5 (maspin), and TNF and downregulated 8 genes such as CCNE1, AKT, ANGPT1, TEK, TGFBR1, MMP9, U-PA, and S100A4. These results indicate that ATA could exert antitumor effects through activating JNK/MAPK and suppressing AKT signal transduction pathways and that ATA might be a potent anticancer agent.

## 1. Introduction

Hepatocellular carcinoma (HCC), the major histological subtype of primary liver cancer, is a refractory malignancy with a high incidence and large mortality [1]. Although great progress in HCC treatment has been achieved during the past three decades, patients in advanced and end stages continue to experience a poor prognosis owing to the paucity of an effective and tolerable systemic chemotherapy strategy [2]. Because monotarget agents fail to completely treat the multifold molecular pathogenesis of HCC and combination regimens tend to cause additional adverse effects, it is essential to develop multitarget compounds that can rectify several aberrant signaling pathways concurrently so as to not only improve the efficacy but also minimize the toxicity [3–5]. With the advancement of techniques in extraction, isolation, and identification of compounds from plants, scientists started to search for antitumor components from herb medicine [6, 7].

**Figure 1:**
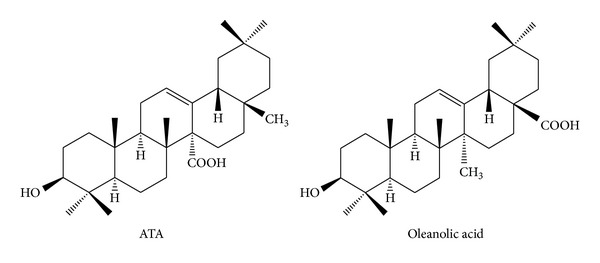
Chemical structures of 3**β**-hydroxy-12-oleanen-27-oic acid (ATA) and oleanolic acid.

The rhizome of *Astilbe chinensis* (Maxim.) Franch. et Savat. (Saxifragaceae) has been used for headache, arthralgia, chronic bronchitis, and stomachalgia in traditional Chinese medicine and several forms of cancers in the folk medicine [8]. In the previous investigation, the triterpenoid fraction from the rhizomes of *A. chinensis* was found to exhibit distinctive antitumor effect *in vivo* [9]. Bioassay-directed purification of this fraction afforded five cytotoxic oleanane triterpenoids [10, 11]. Among five triterpenoids, 3**β**-hydroxy-12-oleanen-27-oic acid (ATA, Figure 1) was a main antitumor active compound. ATA has been proved to inhibit the proliferation of human ovarian carcinoma cells (HO-8910), human cervical squamous carcinoma cells (HeLa), human leukemic cells (HL-60), and human colorectal carcinoma (COLO-205) and induce COLO-205 and HeLa cells into apoptosis *in vitro* [12, 13] as well as to inhibit the growth of transplanted S180 sarcoma and H22 hepatoma in mice [14]. ATA was structurally very similar to oleanolic acid (Figure 1), a well-known hepatinica [15], with the only difference being interchange of the carboxyl and methyl groups at the C-14 and C-17 positions. Our previous experiments also indicated that human hepatoma HepG2 cell was proved more sensitive to ATA than HeLa cell and COLO-205 cell. Therefore, the current experiments were designed to investigate the antiproliferative and antimotility/invasion activity of ATA on human hepatoma HepG2 cells *in vitro* and its inhibitory effect on pulmonary metastasis of B16-F10 melanoma* in vivo*, and explore its molecular mechanisms of action using a high-throughput Cancer Pathway Finder PCR Array.

## 2. Material and Methods

### 2.1. Reagents

3-(4,5-Dimethylthiazol-2-yl)-2,5-diphenyltetrazolium bromide (MTT) and acridine orange (AO) were purchased from Sigma Chemical Co., Saint Louis, MO, USA; RPMI 1640 medium was from Gibco, Grand Island, NY, USA. Hematoxylin-eosin (HE) staining kit was from Amresco, Solon, OH, USA. Cell cycle staining solution (DNA staining solution with Propidium iodide (PI) and RNase A) was purchased from MultiSciences Biotech Co. Ltd., Hangzhou, Zhejiang, China. JC-1 mitochondrial potential sensors, Trizol, RNase-free DNase, and Superscript III RNase Reverse Transcriptase were purchased from Invitrogen, Carlsbad, CA, USA. RT² Profiler Human Cancer Pathway Finder PCR Array (PAHS-033A) and 2× SuperArray PCR master mix were purchased from SABioscience Corp., Frederick, MD, USA; RNeasy MinElute Cleanup Kit was from Qiagen, Valencia, CA, USA; and other PCR reagents revertAid M-MuLV reverse transcriptase, diethylpyrocarbonate (DEPC), ribonuclease inhibitor, Oligo (dT)_18_, standard DNA marker, and PCR primers were from Shanghai Sangon Biological Engineering Technology and Services Co., Ltd., Shanghai, China; basement membrane matrix was from BD Biosciences, San Jose, CA, USA; anti-p38 MAPK, antiphospho-p38 MAPK (Thr^180^/Tyr^182^), anti-ERK1/2, antiphospho-ERK1/2 (Thr^202^/Tyr^204^), anti-SAPK/JNK, and antiphospho-SAPK/JNK (Thr^183^/Tyr^185^) antibodies were from Cell Signaling, Beverly, MA, USA; actin antibody was from Beyotime, Jiangsu, China. The enhanced chemiluminescence (ECL) kit was purchased from Amersham Life Science, Amersham, UK, USA; X-ray films were from Kodak, Rochester, NY, USA. Fetal bovine serum (FBS) was provided by Hangzhou Sijiqing Corp., Hangzhou, Zhejiang, China. Cyclophosphamide (CY) was provided by Jiangsu Hengrui Company, China.

3**β**-Hydroxy-12-oleanen-27-oic acid (ATA, C_30_H_48_O_3_, Mw: 456.3594, Figure 1) was previously isolated by us from the rhizomes of *A. chinensis* (Saxifragaceae) [10]. The structure of ATA was elucidated by spectroscopic analysis including HR-ESI-MS and two-dimensional NMR spectroscopy and confirmed by single-crystal X-ray diffraction analysis [16]. The purity of ATA was determined to be 98.9% using peak area normalization method by HPLC on a Waters 600E HPLC instrument with a Symmetry C18 column (250 mm × 4.6 mm i.d.; 5 *μ*m particle size), a Waters 2996 PDA detector. The stock solutions in DMSO (Sigma) were prepared and diluted as desired with RPMI 1640 medium. The final concentration of DMSO in the assays was less than 0.1% in all experiment and did not show any detectable effect on cell growth or apoptosis.

### 2.2. Cell Culture and Animals

Human hepatoma HepG2 cells and murine melanoma B16-F10 cells were purchased from the Institute of Cell Biology, Shanghai Institute for Biological Sciences, China, and maintained in RPMI-1640 complete medium supplemented with 100 IU/mL penicillin, 100 *μ*g/mL streptomycin, and 10% (v/v) FBS at 37°C under humidified air with 5% CO_2_.

Male C57BL/6 mice (5-6 weeks old) were purchased from Shanghai Experimental Animal Center of Chinese Academy of Sciences (Certificate no. SCXK 2007-0005, Shanghai, China). Rodent laboratory chow and tap water were provided *ad libitum* and maintained under controlled conditions with a temperature of 24 ± 1°C, humidity of 50 ± 10%, and a 12/12-h light/dark cycle. All the procedures were in strict accordance with the PR China legislation on the use and care of laboratory animals and with the guidelines established by the Institute for Experimental Animals of Zhejiang University and were approved by the university committee for animal experiments.

### 2.3. Cell Viability Assay (MTT)

Cell viability was measured by a MTT assay [11]. In brief, HepG2 cells were seeded at 1 × 10^4^ cells per well in a 96-well flat-bottom plate. After 24 h incubation, the various concentrations of ATA or RPMI 1640 medium were added into each well and these cells were incubated at 37°C for the indicated time. Each concentration was repeated four wells. Four h prior to incubation end, 50 **μ**L of MTT solution (2 mg/mL) was added to each well and incubated further for 4 h. To each well, 200 *μ*L of a DMSO working solution (192 *μ*L DMSO with 8 *μ*L 1 N HCl) was added, and the absorbance was evaluated in an ELISA reader at 570 nm with a 630 nm reference after 15 min. The inhibitory rates and 50% inhibitory concentrations (IC_50_) values towards cell proliferation were calculated by NDST software. Each test was performed in triplicate.

### 2.4. Fluorescence Microscope Observation

HepG2 cells were seeded at 1 × 10^5^ cells/mL into 24-well plates (Nunc) and then incubated at 37°C in a humidified atmosphere with 5% CO_2_. After 24 h, the cells were treated without or with ATA for 24 h and 48 h, respectively. After being washed twice with phosphate buffer saline (PBS), cells were stained with 100 *μ*L acridine orange solution (20 **μ**g/mL) for 30 min, and then visualized using Olympus fluorescence microscope (Olympus, Japan) with 488 nm stimulation and 500–520 nm emission.

### 2.5. Measurement of Mitochondrial Transmembrane Potentials **(**Δ*ψ*_*m*_ **)**

Δ*ψ*_*m*_ was evaluated by JC-1 staining [17]. After being treated with different concentrations of ATA for 24 and 48 h, HepG2 cells were washed twice with PBS and incubated with 500 *μ*L of JC-1 staining solution (5 **μ**g/mL) at 37°C for 30 min. The stained cells were rinsed twice with PBS and resuspended in medium. The Δ*ψ*_*m*_ changes were visualized by the relative intensity of dual emissions from mitochondrial JC-1 monomers (green fluorescence) or aggregates (red fluorescence) using Olympus fluorescent microscope under argon-ion 488 nm laser excitation. Meanwhile, the staining fluorescence of individual cell was analyzed with a FACSCalibur flow cytometer. JC-1 was excited by an argon laser (488 nm) and green (530 nm)/red (>570 nm) emission fluorescence was collected simultaneously. Data were analyzed using CellQuest software (BD Biosciences, San Jose, CA, USA).

### 2.6. Cell Cycle Assay

After being treated without or with ATA at the different concentrations for 24 h, HepG2 cells were harvested and washed twice with PBS and then stained with cell cycle staining solution for 30 min at room temperature in dark. Analysis of cell cycle distribution was performed by a FACScan flow cytometer using CellQuest software (BD Biosciences, San Jose, CA, USA).

### 2.7. Cell Adhesion Assay

The efficiency of tumor cell adhesion was determined by measuring the number of cells that attached to wall. HepG2 cells were adjusted to a final concentration of 2 × 10^5^ cells/mL with various concentrations of ATA and seeded into 24-well plates (Nunc). After incubation for 2 and 4 h, nonadherent cells were rinsed off with PBS three times, and the remaining cells were visualized by using an inverted microscope (IX51; Olympus, Japan). A total of 5 random fields were counted for each filter and the images were analyzed using the Image Pro Plus 5 software (Media Cybernetics, Silver Spring, MD., USA). Experiments were performed independently at least three times.

### 2.8. Cell Scratch Assay

Cell scratch assay was taken as reported [18]. Briefly, HepG2 cells (1.5 × 10^5^ cells/well) were seeded into 24-well plate for 24 h. The confluent monolayers were starved with serum-free medium for 8 h, scratched with a 1 mL pipette, and washed 3 times with PBS. Then cells were incubated in serum-free medium containing various concentrations of ATA. Photographs were taken at 0, 24, 36, and 48 h after scratching. Cell migratory ability was determined by measuring the distance between the wound edges in the photographs. The width of the wound was measured using Image Pro Plus 5 Software. The values were the mean for 15 fields from 3 independent cultures.

### 2.9. Transwell Assay

The cell invasion assay was carried out using Transwell Boyden chamber with 8 *μ*m pore filter inserted in 24-well plates [19]. Briefly, the surface of the filter was coated with 40 *μ*L ice-cold matrigel (1 : 10, diluted in RPMI 1640) at 37°C overnight and 700 **μ**L RPMI 1640 medium containing 10% FBS was added to each lower well. 5 × 10^4^ cells in 100 **μ**L RPMI 1640 were loaded into each upper well along with various concentrations of ATA. After incubation at 37°C for 16 h, nonmigrating cells on the upper surface of the filter were removed with a cotton swab. Migrating cells on the lower surface of the filters were fixed with 4% paraformaldehyde and stained with hematoxylin and eosin (HE). The cells were visualized by using an inverted microscope (IX51; Olympus, Japan). A total of 5 random fields were counted for each filter and the images were analyzed using the Image Pro Plus 5 software. The rate of invasion was calculated as migrated cell treated per migrated cells of the control. Experiments were performed independently at least three times.

### 2.10. *In Vivo* Efficacy Study

C57BL/6 mice were injected with B16-F10 melanoma cells (5 × 10^5^ cells/mouse) in the tail veins for 0.05 mL per mouse. Twenty-four h later, mice were divided into five groups, each consisting of ten mice. The inoculated mice were administered per os (*p*.*o*.) with ATT at the doses of 20, 40, and 60 mg/kg for 10 days or injected intraperitoneally (i.p.) with CYT twice at a dose of 50 mg/kg every other day. Model control groups received the same volume of saline. The dose volume was 0.2 mL/10 g body weight. A normal control group without medicine administration and tumor inoculation was also used in this experiment. On day 14 after the tumor cells' challenge, mice were weighed and sacrificed by cervical dislocation. The lung tissues were collected, weighted, and fixed with Bouin's solution. The number of tumor colonies in lung was counted under anatomic microscope (×10). The inhibitory rate (%) = [(*C* − *T*)/*C*] × 100%, where *C* is the average number of tumor colonies of the model control group; *T* is the average number of tumor colonies of medicine groups.

### 2.11. PCR Array Analysis

Quality control of RNA samples, synthesis of cDNA, and real-time RT-PCR arrays were performed as described [20]. All genes represented by the array showed a single peak on the melting curve characteristic to the specific products. Data analysis of gene expression was performed using Excel-based PCR Array Data Analysis Software provided by manufacturer (Qiagen). Fold changes in gene expression were calculated using the ΔΔCt method, and five stably expressed housekeeping genes (*β*2 microglobulin, hypoxanthine phosphoribosyltransferase 1, ribosomal protein L13a, GAPDH, and *β*-actin) were used for the normalization of the results.

### 2.12. RT-PCR Analysis

After incubation with or without ATA, the cells were subjected to Trizol reagent and the total RNA was isolated according to the manufacture's protocol, and reverse transcription and amplification were performed as previously [21]. Then amplification was carried out in a total volume of 20 *μ*L containing 0.5 *μ*L (20 *μ*M) of each specific primer (Table 1), 2 *μ*L of 10 × PCR buffer, 1.2 *μ*L of MgCl_2_ (25 mM), 0.5 *μ*L of dNTP (10 mM), 1 *μ*L of transcribed cDNA, and 0.25 *μ*L of Taq DNA polymerase. PCR was performed for multiple cycles using a PTC-200 thermal cycler (Bio-Rad Laboratories, Inc., USA) with the following program of denaturation at 94°C for 1 min, annealing for 50 s, and elongation at 72°C for 0.5 min. The specific primers, amplified cycles, and annealing temperature of each gene were listed in Table 1. Semiquantitative RT-PCR was performed using *β*-actin as an internal control to normalize gene expression for the PCR templates. The PCR products were analyzed by electrophoresis on a 1.5% agarose gel containing goldview (5 *μ*L/100 mL), and the amplified bands were visualized and photographed using JS-680B Gel Documentation and Analysis System (Shanghai Peiqing Science and Technology Co., Ltd, China). The size of the amplified fragments was determined by comparison with a standard DNA marker.

### 2.13. Western Blot Analysis

After being treated with or without ATA for dedicated times, HepG2 cells were collected and lysed with iced-cold RIPA buffer (1% NP-40, 50 mM Tris-base, 0.1% SDS, 0.5% deoxycholic acid, 150 mM NaCl, and pH 7.5) supplemented with phenylmethylsulfonyl fluoride (10 mg/mL), leupeptin (17 mg/mL), and sodium orthovanadate (10 mg/mL). The protein contents in the supernatant were measured with the BCA protein assay kit. The denatured proteins (30 *μ*g) were separated on 10–12% sodium dodecyl sulfate polyacrylamide gel electrophoresis (SDS-PAGE) and transferred to PVDF membrane. After blocking the membrane with 5% skim milk in Tween-20 containing Tris buffered saline (TTBS) (20 mM Tris-HCl (pH 7.6), 150 mM NaCl, 0.1% Tween-20) for 2 h at 37°C, the blot was incubated with rabbit monoclonal antibody anti-p38 MAPK, antiphospho-p38 MAPK (Thr^180^/Tyr^182^), anti-ERK1/2, antiphospho-ERK1/2 (Thr^202^/Tyr^204^), anti-SAPK/JNK, antiphospho-SAPK/JNK (Thr^183^/Tyr^185^), and mouse monoclonal antibody anti-*β*-actin in TTBS containing 5% skim milk overnight at 4°C. Subsequently, the membranes were washed with TTBS and incubated with an appropriate secondary antibody (horseradish peroxidase-conjugated goat anti-mouse or anti-rabbit IgG) for 2 h. After washing the membrane with TTBS three times for 5 min, the signal was visualized with ECL Detection Kit and exposed the membranes to X-ray films. The band density for the target protein in each sample was measured with JS-680B Gel Documentation and Analysis System and normalized to **β**-actin expression.

### 2.14. Statistical Analysis

Data were expressed as mean ± SD and examined for their statistical significance of difference with ANOVA and a Tukey post hoc test. *P* values less than 0.05 were considered to be statistically significant.

## 3. Results

### 3.1. Effects of ATA on Viability and Morphology of HepG2 Cells

**Figure 2:**
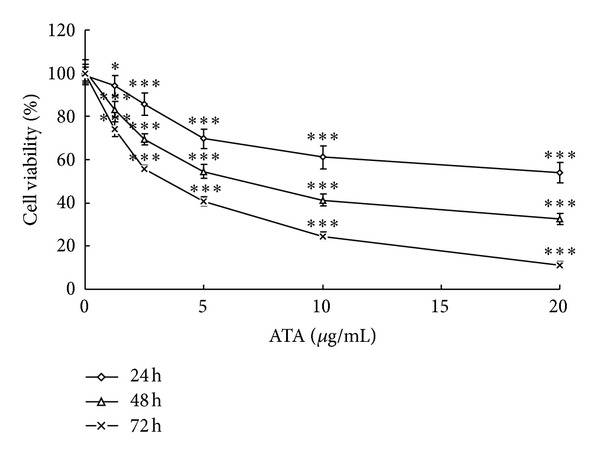
Effect of 3**β**-hydroxy-12-oleanen-27-oic acid (ATA) on the viability of HepG2 cells. HepG2 cells were treated with ATA at indicated concentrations (0–20 **μ**g/mL) of for 24, 48, and 72 h, respectively, and the results were expressed by percentages of surviving cells over untreated control cells using MTT assays. The values are presented as mean ± SD for three independent experiments. Significant differences with 0 **μ**g/mL were designated as *P < 0.05 and ***P < 0.001.

**Figure 3:**
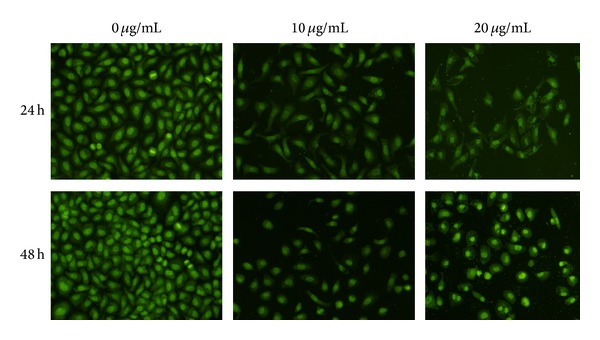
Morphological change of HepG2 cells after treatment with 3**β**-hydroxy-12-oleanen-27-oic acid (ATA) for 24 and 48 h. Morphological changes were visualized under a fluorescence microscope after acridine orange. The figures shown were representative of three independent experiments.

The cytotoxic effect of ATA on HepG2 cells was determined using MTT assay. As shown in Figure 2, ATA significantly inhibited the growth of HepG2 cell in dose- and time-dependent manners. The morphology of HepG2 cells after 24 and 48 h treatment with ATA was also observed by AO staining under fluorescence microscope, and the results were shown in Figure 3. After staining with AO, the DNA in the nucleus of control cells had homogeneously *Kelly* fluorescence, while ATA-treated cells showed typical apoptotic features characterized by volume reduction, chromatin condensation, nuclear fragmentation with densely *Kelly* fluorescence stain, and appearance of apoptotic bodies.

### 3.2. ATA Reduced Mitochondrial Transmembrane Potential (Δ*ψ*_*m*_) of HepG2 Cells

**Figure 4:**
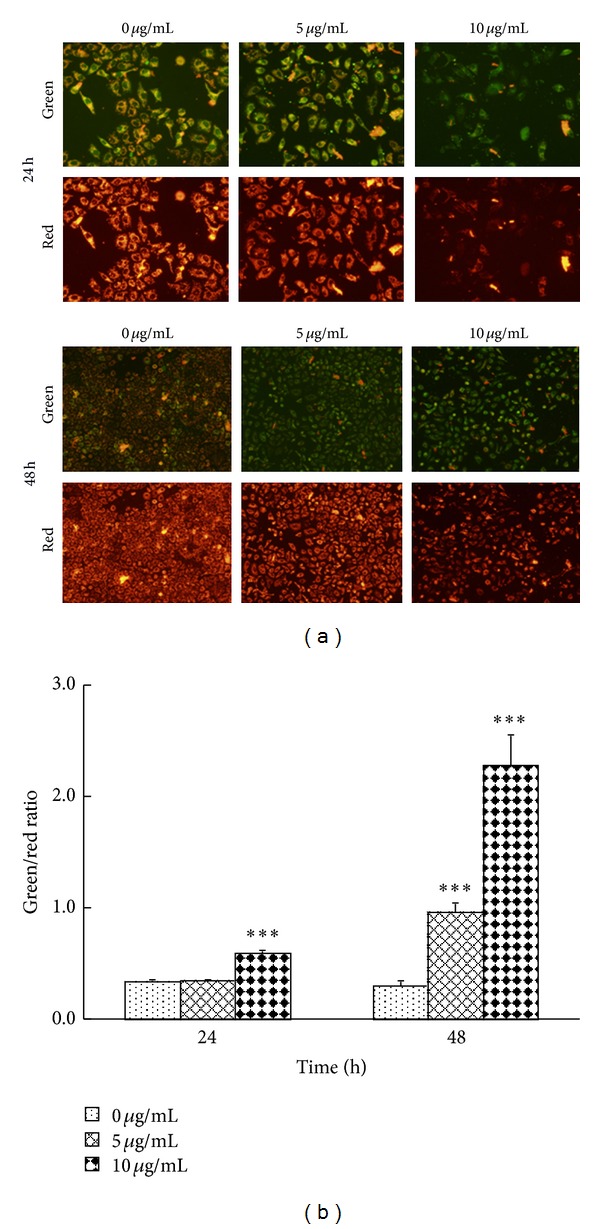
Effect of 3**β**-hydroxy-12-oleanen-27-oic acid (ATA) on the mitochondrial transmembrane potential (Δ*ψ*_*m*_) in HepG2 cells. HepG2 cells were treated with ATA at the concentrations of 0, 5, and 10 *μ*g/mL for 24 and 48 h. (a) Fluorescence changes were visualized under a fluorescence microscope after JC-1 staining. The figures shown were representative of three independent experiments. (b) The staining fluorescence was determined by flow cytometry, and the results were expressed by the ratio of green and red fluorescence. The values are presented as mean ± SD for three independent experiments. Significant differences with 0 **μ**g/mL were designated as ***P < 0.001.

The reduction of Δ*ψ*_*m*_ plays a marked role in early apoptotic cells. JC-1 is an ideal fluorescent probe to detect the change of Δ*ψ*_*m*_, which exhibit red fluorescence as JC-1 aggregates in mitochondrial matrix under high mitochondrial potential but green fluorescence as monomeric form under low potential. To study the potential effects of ATA on the mitochondrial apoptotic pathway, the change of Δ*ψ*_*m*_ in HepG2 cells treated with ATA was measured with JC-1 staining. As shown in Figure 4(a), compared to control cells, the intension of green fluorescence was markedly increased, while that of red fluorescence was highly decreased dose-dependently in ATA-treated HepG2 cells, suggesting that ATA reduced the Δ*ψ*_*m*_ in HepG2 cells in dose- and time-dependent manners. Meanwhile, in order to quantitatively analyze the changes of Δ*ψ*_*m*_ after ATA treatment, cells were determined using flow cytometry and the relative changed values were presented with the ratio of green to red fluorescence. As shown in Figure 4(b), the ratio of green to red fluorescence in HepG2 cells treated with ATA significantly increased, which was consistent with the observation under fluorescence microscope. These results suggest that ATA could decrease the Δ*ψ*_*m*_ without altering plasma membrane permeability in HepG2 cells.

### 3.3. ATA Arrested HepG2 Cells in G_2_/M Phase

**Figure 5:**
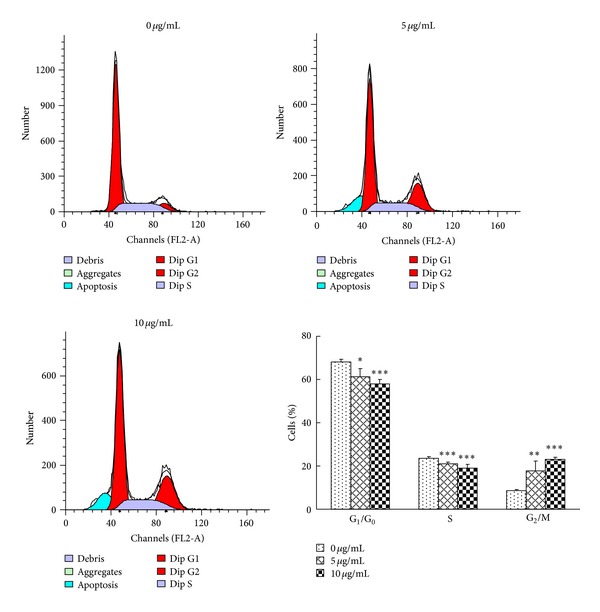
Effect of 3**β**-hydroxy-12-oleanen-27-oic acid (ATA) on cell cycle distribution in HepG2 cells. After treatment with ATA at the concentrations of 0, 5, and 10 **μ**g/mL for 48 h, the cells were collected and stained with propidium iodide (PI), with fluorescence intensities measured by flow cytometry. The histogram demonstrated the percentage of cells in various phase in HepG2 cells treated with ATA. The values are presented as means ± SD for three independent experiments. Significant differences with 0 *μ*g/mL were designated as *P < 0.05, **P < 0.01, and ***P < 0.001.

After treatment with ATA at 5 and 10 *μ*g/mL for 48 h, the obvious changes in cell-cycle distribution of HepG2 cells were characterized by a decrease of the G_0_/G_1_- and S-phase and an increase of the G_2_/M-phase cells in a dose-dependent manner (*P* < 0.05, *P* < 0.01, or *P* < 0.001), suggesting that ATA suppress cell proliferation associated with cell-cycle arrest in the G_2_/M phase (Figure 5).

### 3.4. ATA Suppressed HepG2 Cell Adhesion

Because the disruption of the cell-substratum and cell-cell adhesion plays an important role in tumor metastasis, we first investigated whether ATA could affect the ability of tumor cells to adhere to wall. HepG2 cells treated with ATA showed a dose- and time-dependent decrease in cell adhesion. ATA induced a decrease in the proportion of adherent cells, with being 64.09%, 48.86%, and 34.65% for 2 h and 56.97%, 38.95%, and 33.98% for 4 h at the concentrations of 5, 10, and 20 **μ**g/mL, respectively (Table 2).

### 3.5. ATA Inhibited Migration of HepG2 Cells

**Figure 6:**
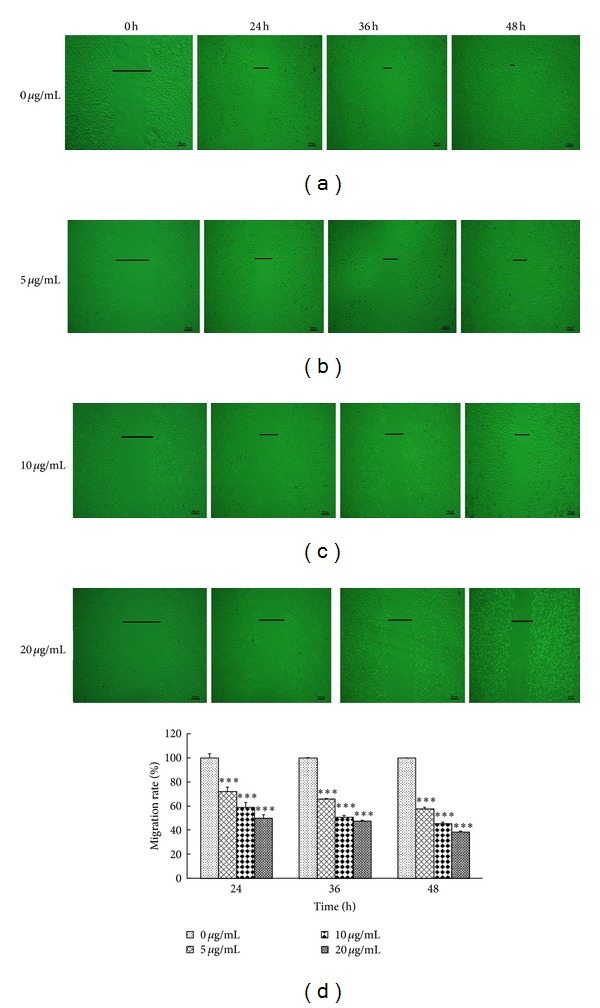
Effect of 3**β**-hydroxy-12-oleanen-27-oic acid (ATA) on the migration of HepG2 cells. HepG2 cells were plated in a 24-well plate and the confluent monolayers were wounded and then incubated with ATA at the concentrations of 0, 5, 10, and 20 **μ**g/mL in serum-free medium. At 0, 24, 36, and, 48 h after wounding; the cells were photographed under an inverted microscope. The histogram demonstrated the migration rate expressed as a percentage of control (0 **μ**g/mL). The values are presented as means ± SD for three independent experiments. Significant differences with 0 **μ**g/mL were designated as ***P < 0.001.

As tumor cell migration is an essential process in cancer development and metastasis, we used a scratch wound assay to determine the effect of ATA on HepG2 cell migration. As shown in Figure 6, the control cells migrated along the edges of the wound, showed a large-scale migration, and fully recovered 48 h after scratching, whereas the inhibition of cell flattening and spreading was observed in the presence of ATA. After treatment with ATA at the concentrations of 5, 10, and 20 **μ**g/mL, the average migration rates were 72%, 59%, and 50% for 24 h; 65%, 50%, and 47% for 36 h; and 57%, 45%, and 38% for 48 h, respectively. These results suggested that ATA significantly inhibit the migratory ability of HepG2 cells in concentration- and time-dependent manner (*P* < 0.001).

### 3.6. ATA Suppressed Invasion of HepG2 Cells

**Figure 7:**
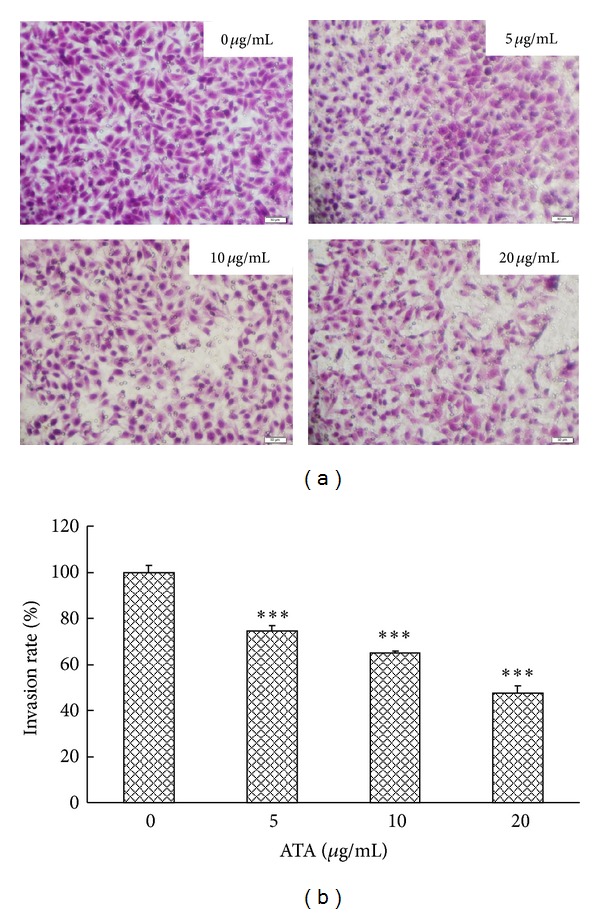
Effect of 3**β**-hydroxy-12-oleanen-27-oic acid (ATA) on the invasion of HepG2 cells. (a) HepG2 cells seeded in Transwell Boyden Chambers were treated by 0, 5, 10, and 20 *μ*g/mL ATA for 16 h, and the cells invading through the matrigel were photographed after HE staining. (b) The invasion rate of HepG2 cells was expressed as the percentage of invading cells compared with the control. The values are presented as means ± SD for three independent experiments. Significant differences with 0 *μ*g/mL were designated as ***P < 0.001.

Transwell Boyden chamber assay was performed to determine the effect of ATA on the motility and invasive of HepG2 cells. As shown in Figure 7(a), incubation of control HepG2 cells in the chamber for 16 h resulted in large-scale migration of cancer cells to the lower side of the filter. In contrast, treatment with ATA caused a dose-dependent inhibition of HepG2 invasion. Compared to the control cell, the average migration rates of HepG2 cell treated ATA at the concentrations of 5, 10, and 20 *μ*g/mL for 16 h were 74.3%, 64.9%, and 47.6%, respectively (Figure 7(b)). The result indicated that treatment with ATA induced a significant decrease in the invasiveness of HepG2 cells (*P* < 0.001).

### 3.7. ATA Inhibited Pulmonary Metastasis *In Vivo*

The experimental lung metastasis models of B16-F10 melanoma in mice were used to investigate the inhibitory effects of ATA on tumor metastasis. As shown in Table 3, the number of tumor colonies in lung and lung weight in each ATA group was less than those in control group (*P* < 0.05), suggesting that ATA can significantly inhibit the lung metastasis of B16-F10 melanoma in mice.

### 3.8. ATA Regulated Expression of Genes Associated with Apoptosis, Cell Cycle, Angiogenesis, Invasion, and Metastasis of HepG2 Cells

To determine the mechanism involved in the antitumor action of ATA on HepG2 cells, 84 genes related to cell proliferation, apoptosis, cell cycle, angiogenesis, invasion, and metastasis in ATA-treated and untreated HepG2 cells were evaluated for expression using real-time PCR via the RT^2^ Profiler Human Cancer Pathway Finder PCR Array, and the results were shown in Table 4. After treatment with ATA, 17 genes in HepG2 cells exhibited significant change in mRNA expression relative to control (expression ratio showing greater than 2-fold or less than 0.5-fold difference compared with the control group). Among the differentially expressed genes, there were 9 genes upregulated and 8 genes downregulated.

Expression of mRNA for several factors involved in cell-cycle control and DNA damage repair including cyclin-dependent kinase inhibitor 1A (CDKN1A, p21) and Mdm2 p53 binding protein homolog (MDM2) were upregulated (4.86- and 3.56-fold, resp.), whereas that of cyclin E1 (CCNE1) was lowered significantly by 2.71-fold by ATA. Transcriptional levels of proapoptotic genes such as CASP8 and FADD-like apoptosis regulator (CFLAR, CASPER) and tumor necrosis factor receptor superfamily, member 10b, (TNFRSF10B, DR5) were upregulated by ATA (2.41- and 2.7-fold, resp.). The mRNA levels of signal-transduction molecules and transcription factors such as V-akt murine thymoma viral oncogene homolog 1 (AKT) were downregulated (2.29-fold), whereas C-Jun oncogene (c-Jun) was upregulated (8.55-fold) after treatment with ATA. Transcriptional levels of several factors related to angiogenesis, such as angiopoietin 1 (ANGPT1), TEK tyrosine kinase, endothelial (TEK), and transforming growth factor, beta receptor 1 (TGFBR1), were downregulated (4.19-, 7.45-, and 4.08-fold, resp.), whereas interleukin 8 (IL-8), thrombospondin 1 (THBS1), Serpin peptidase inhibitor, clade B (ovalbumin), member 5 (SERPINB5, PI5, maspin), and tumor necrosis factor (TNF) were upregulated (13.09-, 2.17-, 2.06-, and 8.20-fold, resp.) after being treated with ATA. The mRNA levels for several molecules involved in invasion and metastasis, including matrix metallopeptidase 9 (MMP9), plasminogen activator, urokinase (PLAU, u-PA), and S100 calcium binding protein A4 (S100A4), were lowered by ATA (2.55-, 4.88-, and 5.70-fold, resp.). ATA, therefore, may exert its antitumor effect on HepG2 cells through altering the expression levels of these genes.

### 3.9. Validation of Cancer Pathway Finder PCR Array Results by RT-PCR

**Figure 8:**
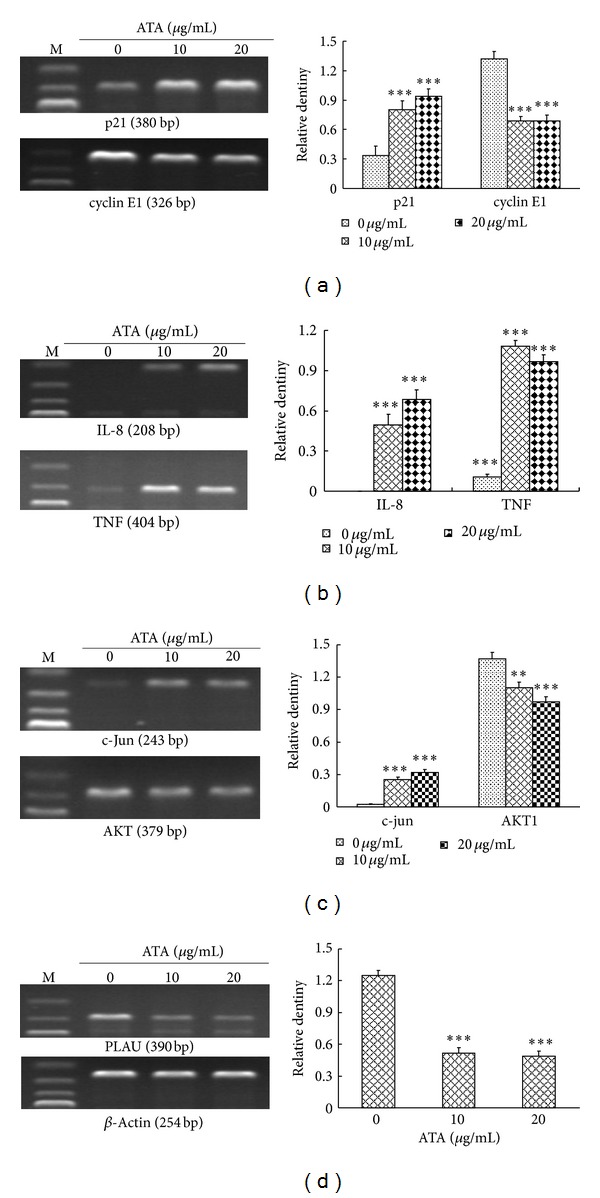
RT-PCR validation of PCR array results. After cells were treated by 0, 10, and 20 *μ*g/mL ATA for 16 h, expression changes of seven genes which were upregulated (p21, IL-8, TNF, and c-Jun) or downregulated (cyclinE1, AKT, and PLAU) in PCR array analysis detected by RT-PCR using specific primers as described in the text. The housekeeping gene *β*-actin was used as endogenous control. The figures shown were representative of three independent experiments. The values are presented as mean ± SD for three independent experiments. Significant differences with 0 **μ**g/mL were designated as **P < 0.01 and ***P < 0.001.

To confirm the validity of the PCR array results, semiquantitative RT-PCR was undertaken for 7 putative differentially expressed genes including 4 upregulated genes p21, IL-8, TNF, and c-Jun and 3 downregulated genes Cyclin E1, AKT, and PLAU. As shown in Figure 8, after HepG2 cells were treated with ATA, the expression levels of p21, IL-8, TNF, and c-Jun mRNA were increased, whereas those of Cyclin E1, AKT, and PLAU were significantly decreased in a concentration-dependent manner compared with the control (*P* < 0.01 and *P* < 0.001). Differential expression of selected genes was considered to be validated as the similar results between RT-PCR and PCR array data.

### 3.10. Involvement of MAPK Pathway in HepG2 Cells Treated with ATA

**Figure 9:**
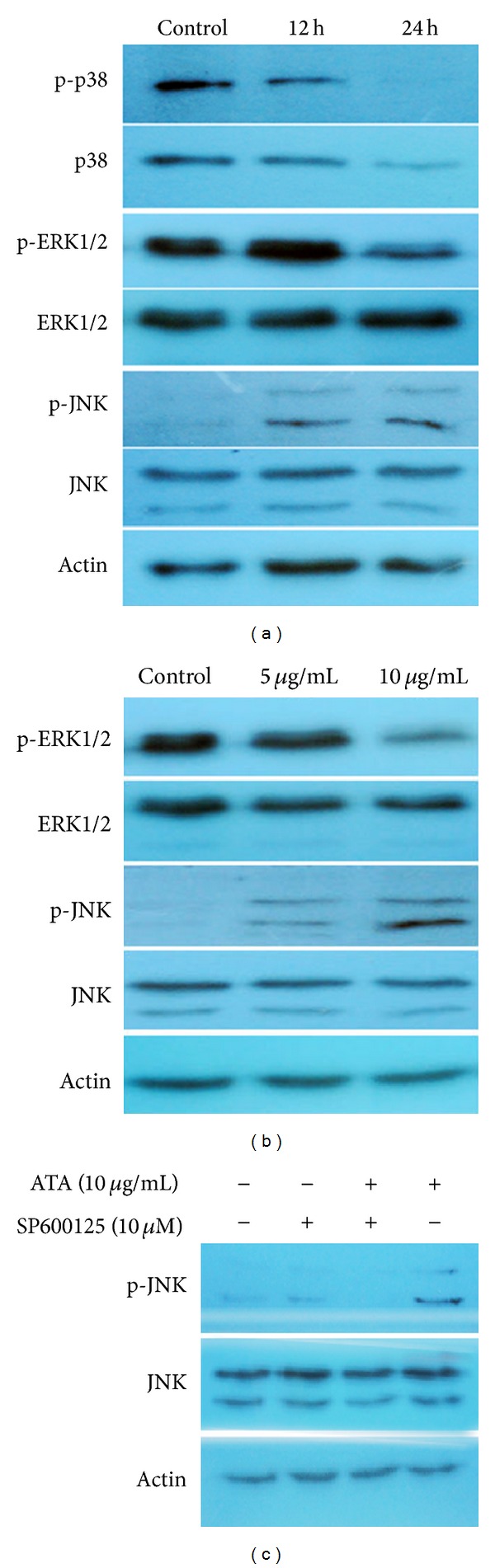
Effect of 3**β**-hydroxy-12-oleanen-27-oic acid (ATA) on MAPK signal pathway in HepG2 cells detected by Western blot. (a) HepG2 cells were treated with 10 **μ**g/mL of ATA for 0, 12, and 24 h, respectively. Then the cells were harvested and the total protein was separated by SDS-PAGE. Total and phosphorylated expression levels of the MAPK family members (JNK, p38, ERK1/2, p-JNK, p-p38, and p-ERK1/2) were examined. (b) HepG2 cells were treated with 5 and 10 *μ*g/mL of ATA for 24 h, respectively. (c) HepG2 cells were treated with the JNK inhibitor SP600125 (10 *μ*M) for 1 h before treatment with 10 **μ**g/mL of ATA for 24 h and p-JNK expression level was determined. Actin was used as an internal control. The figures shown were representative of three independent experiments.

The expression level of c-Jun mRNA in HepG2 cells was significantly upregulated by ATA, suggesting that MAPK pathway may be involved in the anti-tumor effect of ATA. Therefore, the expression levels of total and phosphorylated protein in the MAPK family (JNK, p38, ERK1/2, p-JNK, p-p38, and p-ERK1/2) were examined by Western blot, and the results were shown in Figures 9(a) and 9(b). Although the levels of total JNK and ERK1/2 were as steady as actin in HepG2 cells treated with ATA, the phosphorylation levels of JNK and ERK1/2 were upregulated and downregulated in dose- and time-dependent manners, respectively. However, the phosphorylation of p38 in HepG2 cells was hardly affected by ATA as the change trend was almost the same as total p38. These results indicated that JNK activation and ERK1/2 suppression, but not p38, played critical roles in ATA-induced inhibition in HepG2 cells. Furthermore, the effect of JNK inhibitor SP600125 was applied to confirm the activation of JNK pathway involved in ATA-treated HepG2 cells. As shown in Figure 9(c), the incubation with 10 *μ*M SP600125 for 1 h before ATA treatment significantly suppressed the JNK phosphorylation induced by 10 *μ*g/mL of ATA. These results indicated that activation of JNK pathway may be the key regulator during ATA-induced antitumor effect.

## 4. Discussion

3**β**-Hydroxy-12-oleanen-27-oic acid (ATA, Figure 1) was the main antitumor active compound in the rhizomes of *A. chinensis,* a well-known anti-tumor herb in Chinese folk. ATA has been proved to possess antitumor effect both *in vitro* and *in vivo* [12–14]. In this study, we further investigated the antiproliferative, antiapoptosis, and antimotility/invasion activity of ATA using HepG2 cells *in vitro* and B16-F10 cells *in vivo* and explored its molecular mechanisms of action.

MTT assay showed that ATA could significantly inhibit the growth of HepG2 cells in concentration- and time-dependent manner (Figure 2). The morphology of HepG2 cells treated with ATA presented typical apoptotic features (Figure 3). The Δ*ψ*_*m*_ of HepG2 cells was markedly decreased by ATA (Figure 4). These suggested that ATA could induced HepG2 cells into apoptosis, which was in agreement with the results reported previously [12, 13].

The effect of ATA on HepG2 cell cycle was also investigated. It is interesting to note that ATA could affect different phases of the cell cycle in different cancer cells. We previously reported that ATA resulted in HeLa and COLO-205 cell growth arrest at G_0_/G_1_ phase [12, 13]. In this study, however, ATA was found to arrest HepG2 cells in G_2_/M phase (Figure 5). These results suggested that ATA inhibits tumor cell growth via different mechanisms or different cellular targets in different cell lines. The interesting phenomenon was also found to occur in the traditional Chinese medicine *Scutellaria baicalensis* [22]. *S. baicalensis* could cause G_1_ phase arrest in LNCaP cells [23] but G_2_/M phase arrest for squamous cells, PC-3, and HepG2 cells [22, 24].

Invasion and metastasis, characteristic of cancer, were considered as the key factors that threatened cancer clinical treatment into failure and prognosis recurrence, so that inhibitory effects of invasion and metastasis have been the focus of developing new antitumor drugs. However, effects of ATA on invasion and metastasis of tumor cells have not been reported yet. Therefore, we also investigated the effect of ATA on the adhesion, migration, and invasion of HepG2 cells. As shown in Figures 5–7, ATA could significantly inhibit the adhesive, invasive, and metastatic abilities of HepG2 cells.* In vivo* efficacy study revealed that ATA remarkedly suppressed the lung metastasis of B16-F10 melanoma in mice (Table 3), further confirming the antimetastasis actions of ATA.

A full understanding of molecular effect (i.e., gene expression) of a drug is very important for thr evaluation of its efficacy as well as safety (side effect). The Human Cancer Pathway Finder PCR array provided a simple and sensitive tool for profiling the expression of 84 genes related to cell proliferation, apoptosis, cell cycle, angiogenesis, invasion, and metastasis. To further insight into the molecular mechanism of antitumor action of ATA, Cancer Pathway Finder PCR Array was selected for exploring the effect of ATA on 84 genes representative of the six biological pathways in HepG2 cells. The differentially expressed genes induced by ATA in HepG2 cells included 9 upregulated and 8 downregulated genes. This PCR Array results were further verified by RT-PCR assay. In cell cycle-related genes, p21 (CDKN1A) and MRM2 mediating p53 approach were upregulated, but cyclin E1 was downregulated, which was often a sign of G_0_/G_1_ arrest [25]. However, Lee et al. [26] also reported that quercetin blocked U937 cells in G_2_/M phase with downregulation of cyclin E1. It was also said that cyclin E1 could retard the progress of cell cycle but not in G_0_/G_1_ doubtlessly [27]. ATA upregulated the expression level of apoptosis-related genes CFLAR and DR5 in HepG2 cells, thus suggesting its role in the regulation of the extrinsic (death receptor) pathway also at transcriptional level [28].

Angiogenesis is always thought as an essential role in invasion and metastasis of tumor progression by providing powerful material motivation for invasion and convenient metastatic access [29]. And it was also studied that, with restraining angiogenesis, the abilities of invasion and metastasis in mice were reduced effectively [30]. In this study, we observed that after ATA treatment, the expression levels of proangiogenic factors ANGPT1, TEK (Tie-2), and TGFBR1 were lowered, and those of antiangiogenic factor THBS1 and SERPINB5 expression was increased, respectively. Tie-2 is a tyrosine kinase, and its activation promotes vessel assembly and maturation [31]. ANGPT1 is specific ligands of Tie-2. The increased ANGPT1 expression in HeLa cells by transgene promotes the growth of human cervical cancers in mice via promoting tumor angiogenesis [32]. SERPINB5, a member of the serine protease inhibitor family, has been shown to be a potent metastasis suppressor [33]. THBS1 (TSP-1) has been shown to have antiangiogenic properties and inhibit angiogenesis via a direct way that suppresses migration and induces apoptosis of endothelial cells or via an indirect way that inhibits the mobilization of proangiogenic factors and blocks their access to coreceptors on the endothelial cell surface. TGFBR1 (ALK-5) is a transforming growth factor beta receptor 1. Activated TGFBR1 resulted in heterodimerization of SMAD3 with SMAD4, SMAD3/SMAD4 nuclear translocation, activation of TGF-b target genes, and secretion of angiogenic factor TGF-*β* [34]. In addition, in angiogenesis-relative genes, two anticancer and immune regulation factors TNF and IL-8 were upregulated. In order to survive, the metastatic cells had to escape from biological immune system. The increase of TNF and IL-8 was closely related to enhancement of immune response [35] and indicated that the inhibitory effects of ATA on invasion and metastasis were associated with immune regulation. As was reported that overexpressed caveolin-1 could reduce the aggressiveness of human glioblastoma U87MG cells, the expression of IL-8 was also greatly upregulated [36]. It was also reported that the extract of huanglian, a medicinal herb, induced cell growth arrest and apoptosis by upregulation of IFN-*β* and TNF-*α* in human breast cancer cells [37]. ATA also decreased the invasion-related genes MMP-9, PLAU (u-PA), and S100A4. MMP-9 and PLAU were the most important degradation enzymes of extracellular matrix, while degradation of extracellular matrix was a necessary process during tumor invasion, and metastasis [38]. The MMP-9 gene is strongly expressed in invasive HCC [39], and the MMP-9 protein content in HCC is higher than that in the surrounding liver parenchyma. The activity of MMP-9 is tightly controlled, with regulation occurring primarily at the transcriptional level. PLAU is reported to play an important role in tumor progression and invasion with the aid of the uPA receptor (uPAR) that is expressed on the tumor cell surface [40]. The calcium-binding protein S100A4 also plays an important role in the metastatic journey of tumor cells by influencing several steps in the metastatic cascade, including migration, invasiveness, and angiogenesis [41]. S100A4 protein expression is associated with patient outcome in a number of tumor types. Moreover, there is evidence that S100A4 controls the invasive potential of human prostate cancer cells through the regulation of MMP9 expression [42], thus contributing to the invadopodia-mediated proteolytic degradation of ECM components [43]. S100A4 gene suppression significantly decreased the expression of MMP-9 and overexpression of the S100A4 gene significantly increased MMP-9 expression, suggesting that transcriptional activation of MMP-9 is regulated by the S100A4 gene [44].

Among 17 differentially expressed genes induced by ATA in HepG2 cells, 29% of genes were related to cell apoptosis and cell cycle, 12% to signal transduction and transcription factor, and the other 59% to angiogenesis, invasion, and metastasis. Furthermore, the genes with the most significant, differential expression were involved in angiogenesis, invasion, and metastasis (IL-8 and TNF increased 13.09 and 8.2-fold, TEK, S100A4 and PLAU decreased 7.45, 5.70 and 4.88-fold, repectively) or signal transduction molecules and transcription factor (c-Jun up-regulated 8.55-fold). Considering that the number of the genes detected in each pathway of the PCR array was nearly the same (about 13–15 genes), it suggested that compared with the influences on cell apoptosis and cell cycle, the effects of ATA on angiogenesis, invasion, and metastasis of HepG2 cells seemed more significant. Therefore, suppressing invasion, and metastasis could play more important part in antitumor effect of ATA.

The signal transduction cascades play an important regulatory role in cell growth, differentiation, apoptosis, and metastasis [45]. As an important signal transduction factor c-Jun, activated by the mitogen-activated protein kinases (MAPK) family members, was sharply increased (c-Jun increased 8.55-fold), suggesting that MAPK signaling pathway was involved in the anti-tumor effect of ATA. The MAPK serine/threonine kinase superfamily is activated by numerous extracellular stimuli and is involved in signal transduction cascades playing an important regulatory role in cell growth, differentiation, apoptosis, and metastasis [46]. Three major mammalian MAP kinases have been described: ERK1/2 or p44/42 MAPK, c-Jun N-terminal kinase/stress-activated protein kinase (JNK), and p38 MAPK. The diverse MAP kinase members are activated in response to different extracellular stimuli and have distinct downstream targets, thus serving different roles in cellular responses. Therefore, the total and phosphorylated expression levels of the MAPK family members (JNK, p38, ERK1/2, p-JNK, p-p38, and p-ERK1/2) were further detected by Western blot. The results showed that ATA greatly increased the phosphorylation of JNK and decreased the phosphorylation of ERK1/2 but had little effect on phosphorylation of p38. It was reported that high glucose was reported to induce human endothelial cells into apoptosis by sequential activations of JNK but no involvement of ERK1/2 and p38 [47]. It also was reported that photodynamic therapy (PDT) could inhibit the MDR activity by downregulating the expression of P-glycoprotein *via* JNK activation using pheophorbide a (Pa) as the photosensitizer, and Pa-PDT inhibited the growth of MDR hepatoma cells by mitochondrial-mediated apoptosis induction [48]. Meanwhile, another important signal transduction factor AKT was found to be downregulated by ATA treatment, similar to tamoxifen-induced apoptosis [49]. The AKT pathways are critical for angiogenesis and tumorigenesis [50]. By activating AKT signaling, the expression of various angiogenic factors [51], angiogenesis [52], and tumor growth [53] is increased. In fact, combined effect of Akt and MARK signal transduction pathways was universal in the action of antitumor agents. Curcumin induced tumor cells to apoptosis and blocked the cell cycle in G_2_/M phase by enhancing MAPK pathway and inhibiting the activity of AKT [54]. Purified cranberry proanthocyanidins reduced the expression of prosurvival (Bcl-2, MCL-1, and Bcl-xL) and increased levels of proapoptotic (Bax, Bad, and Bid) Bcl family proteins, upregulated the activity of SAPK/JNK MAPK, and downregulated expression or activity of PI3K/AKT/mTOR pathway components [55]. A well-known antitumor agent green tea catechin also acted by affecting MAPK, and PI3K/AKT pathway [56]. Therefore, ATA could induce apoptosis, block in G_2_/M phase, and inhibit invasion and metastasis through activating JNK and suppressing AKT signal transduction pathways together.

In conclusion, ATA could not only induce tumor cells into apoptosis through the activation of both extrinsic and intrinsic pathways and arrest HepG2 cells in G_2_/M phase mediating p53 approach but also suppress the invasion and metastasis abilities of HepG2 cells by altering the expression of angiogenesis-relative genes and downregulating the expression level of MMP-9, PLAU, and S100A4 mRNA. What is more important, these effects of ATA should act through activating JNK/MAPK and suppressing AKT signal transduction pathways.

## Figures and Tables

**Table 1:** Primer sequences and amplified conditions used for RT-PCR.

Gene	Primer sequence	Product size (bp)	Annealing temperature (°C)	Cycles
**β**-actin	5′-CTGTCTGGCGGCACCACCAT-3′	254	54	21
	5′-GCAACTAAGTCATAGTCCGC-3′			
p21	5′-AGTGGACAGCGAGCAGCTGA-3′	380	60	27
	5′-TAGAAATCTGTCATGCTGGTCTG-3′			
c-Jun	5′-ATGGAAACGACCTTCTATGACG-3′	243	60	29
	5′-GTTGCTGGACTGGATTATCAGG-3′			
TNF	5′-TGTAGCCCATGTTGTAGCAAAC-3′	404	54	33
	5′-ACTAGGCAAAGTCGAGATAGTC-3′			
IL-8	5′-GTTTTGCCAAGGAGTGCTAAAG-3′	208	60	29
	5′-AAAACTTCTCCACAACCCTCTG-3′			
PLAU	5′-TTGATTACCCAAAGAAGGAGGA-3′	390	54	33
	5′-TGGTGACTTCAGAGCCGTAGTA-3′			
Cyclin E1	5′-AGTTTGCGTATGTGACAGATGG-3′	326	60	31
	5′-CTGATACCCTGAAACCTTTTGC-3′			
AKT	5′-TCTACAACCAGGACCATGAGAA-3′	379	54	26
	5′-GAGTAGGAGAACTGGGGGAAGT-3′			

**Table 2:** Effects of 3**β**-hydroxy-12-oleanen-27-oic acid (ATA) on the adhesion of HepG2 cells.

ATA (*μ*g/mL)	Adhesive rate (%)	
	2 h	4 h
0	100.00 ± 3.33	100.00 ± 4.94
5	64.09 ± 3.02***	56.97 ± 2.57***
10	48.86 ± 2.26***	38.95 ± 2.69***
20	34.65 ± 1.57***	33.98 ± 0.47***

Adhesive rate was expressed as a percentage of control (0 *μ*g/mL). The values are presented as means ± SD for three independent experiments. Significant differences with 0 *μ*g/mL were designated as ***P < 0.001.

**Table 3:** Inhibitive effect of 3*β*-hydroxy-12-oleanen-27-oic acid (ATA) on pulmonary metastasis of B16-F10 melanoma in mice.

Group	Dose (mg/kg)	Body weight (g)	Lung weight (mg)	No. of tumor colonies	Inhibitory rate (%)
Normal control	—	20.99 ± 2.88	139.86 ± 17.01	—	—
Model control	—	20.09 ± 1.85	212.20 ± 14.85	54.33 ± 11.75	—
CY	50	20.74 ± 2.44	157.84 ± 11.58***	12.00 ± 6.20***	77.91
ATA	20	20.80 ± 2.35	177.97 ± 16.01**	23.38 ± 11.49***	56.96
	40	20.28 ± 2.34	159.43 ± 12.74***	16.29 ± 6.74***	70.03
	60	20.58 ± 2.06	150.64 ± 13.06***	11.80 ± 9.91***	78.28

The values are presented as means ± SD (*n* = 10). Significant differences with model control were designated as **P < 0.01 or ***P < 0.001. CY: cyclophosphamide (positive drug).

**Table 4:** Genes found to be upregulated or downregulated by 3*β*-hydroxy-12-oleanen-27-oic acid (ATA) in HepG2 cells based on PCR array.

Functional gene groups	Symbol	UniGene	RefSeq	Description	Fold
Cell cycle control & DNA damage repair	CDKN1A (p21Waf1)	Hs.370771	NM_000389	Cyclin-dependent kinase inhibitor 1A (p21, Cip1)	**4.86**
	MDM2	Hs.484551	NM_002392	Mdm2 p53 binding protein homolog (mouse)	**3.56**
	CCNE1	Hs.244723	NM_001238	Cyclin E1	**−2.71**

Apoptosis and cell senescence	CFLAR (CASPER)	Hs.390736	NM_003879	CASP8 and FADD-like apoptosis regulator	**2.41**
	TNFRSF10B (DR5)	Hs.521456	NM_003842	Tumor necrosis factor receptor superfamily, member 10b	**2.70**

Signal transduction molecules and transcription factors	c-Jun	Hs.714791	NM_002228	C-Jun oncogene	**8.55**
	AKT	Hs.525622	NM_005163	V-akt murine thymoma viral oncogene homolog 1	**−2.29**

Angiogenesis	IL-8	Hs.624	NM_000584	Interleukin 8	**13.09**
	THBS1	Hs.164226	NM_003246	Thrombospondin 1	**2.17**
	TNF	Hs.241570	NM_000594	Tumor necrosis factor (TNF superfamily, member 2)	**8.20**
	ANGPT1	Hs.369675	NM_001146	Angiopoietin 1	**−4.19**
	TEK (tie-2)	Hs.89640	NM_000459	TEK tyrosine kinase, endothelial	**−7.45**
	SERPINB5 (maspin)	Hs.55279	NM_002639	Serpin peptidase inhibitor, clade B (ovalbumin), member 5	2.06
	TGFBR1 (ALK-5)	Hs.494622	NM_004612	Transforming growth factor, beta receptor 1	**−4.08**

Invasion and metastasis	MMP9 (gelatinase B)	Hs.297413	NM_004994	Matrix metallopeptidase 9	**−2.55**
	PLAU	Hs.77274	NM_002658	Plasminogen activator, urokinase	**−4.88**
	S100A4	Hs.654444	NM_002961	S100 calcium binding protein A4	**−5.70**

Gene alterations after ATA-exposed HepG2 cells were analyzed using quantitative real-time PCR profiling. Only signals that differed from untreated cells by at least 2-fold were considered as significant. Changes are indicated as (+) upregulation and (−) downregulation as compared to control group.
